# Subarachnoid haemorrhage due to intracranial vertebral artery dissection presenting with atypical cauda equina syndrome features: case report

**DOI:** 10.1186/s12883-019-1487-z

**Published:** 2019-10-30

**Authors:** Lloyd Steele, Muhammad Hasan Raza, Richard Perry, Neil Rane, Sophie J. Camp

**Affiliations:** 1 Department of Neurosurgery, Charing Cross Hospital, Imperial College Healthcare NHS Trust, Fulham Palace Rd, Hammersmith, London, W6 8RF UK; 2 Department of Neurology, Charing Cross Hospital, Imperial College Healthcare NHS Trust, London, UK; 3 Department of Interventional Neuroradiology, Charing Cross Hospital, Imperial College Healthcare NHS Trust, London, UK

**Keywords:** Case report, Hypesthesia, Vertebral artery, Subarachnoid hemorrhage, Aneurysm, Dissecting, Headache, Facial nerve

## Abstract

**Background:**

Failing to recognise the signs and symptoms of subarachnoid haemorrhage (SAH) causes diagnostic delay and may result in poorer outcomes. We report a rare case of SAH secondary to a vertebral artery dissection (VAD) that initially presented with cauda equina-like features, followed by symptoms more typical of SAH.

**Case presentation:**

A 55-year-old man developed severe lower back pain after sudden movement. Over the next 5 days he developed paraesthesiaes in the feet, progressing to the torso gradually, and reported constipation and reduced sensation when passing urine. On day six he developed left facial palsy, and later gradual-onset headache and intermittent confusion.

Magnetic resonance imaging of the brain showed diffuse subarachnoid FLAIR hyperintensity, concerning for blood, including a focus of cortical/subcortical high signal in the left superior parietal lobule, which was confirmed by computed tomography. Digital subtraction angiography demonstrated a left VAD with a fusiform aneurysm.

**Conclusion:**

We present a very rare case of intracranial VAD with SAH initially presenting with spinal symptoms. The majority of subsequent clinical features were consistent with a parietal focus of cortical subarachnoid blood, as observed on neuroimaging.

## Background

The reported rates of misdiagnosis of nontraumatic subarachnoid haemorrhage (SAH) in the literature have been significant (5%, [1, 2] 12–20% [3] and 13–43% [4]). Whilst this is most common with lower acuity presentations, [1] initial misdiagnosis may later result in a catastrophic outcome [4].

In this case we describe a rare presentation of SAH secondary to vertebral artery dissection (VAD) that initially presented with spinal symptoms. Only two previous cases of intracranial VAD have been associated with spinal extension of SAH [5, 6].

## Case presentation

A 55-year-old man developed severe lower back pain after heavy lifting. Over the next 5 days he experienced paraesthesiaes in the feet, progressing to the torso, and reduced sensation when passing urine. On day six he presented to hospital after waking and noticing a left facial droop. On presentation he also reported a 3 day history of constipation. There was no history of headache, neck stiffness, or trauma. He was a current smoker (15 cigarettes per day) and drank 70 units of alcohol per week. There was no significant past medical history and he used no regular medications.

His vital signs on admission revealed hypertension (blood pressure 160/92 mmHg) and tachycardia (heart rate 103 beats per minute), but were otherwise within normal limits. Glasgow coma scale was 15. On examination there was left-sided facial weakness (House-Brackmann grade IV) with no forehead sparing (lower motor neurone pattern). The remainder of the cranial nerve examination was normal. Tone was normal and there was no clonus. Pyramidal drift was present in the right upper limb, but muscle power was Medical Research Council grade 5/5 in all muscle groups. Deep tendon reflexes were elicited (normal) apart the ankle reflex, which was absent bilaterally. The plantar response was downgoing bilaterally. Light touch and pain sensation were reduced, but present, to T6 bilaterally - most notably across L4-S1 and S3-S4 (saddle anaesthesia) – and vibration sense was reduced to the ankle bilaterally. Gait was unsteady and the patient was unable to tandem walk. There was no limb ataxia. Anal tone was normal, there was no significant residual volume on urinary catheterisation, and the patient could feel a catheter tug.

**Fig. 1:**
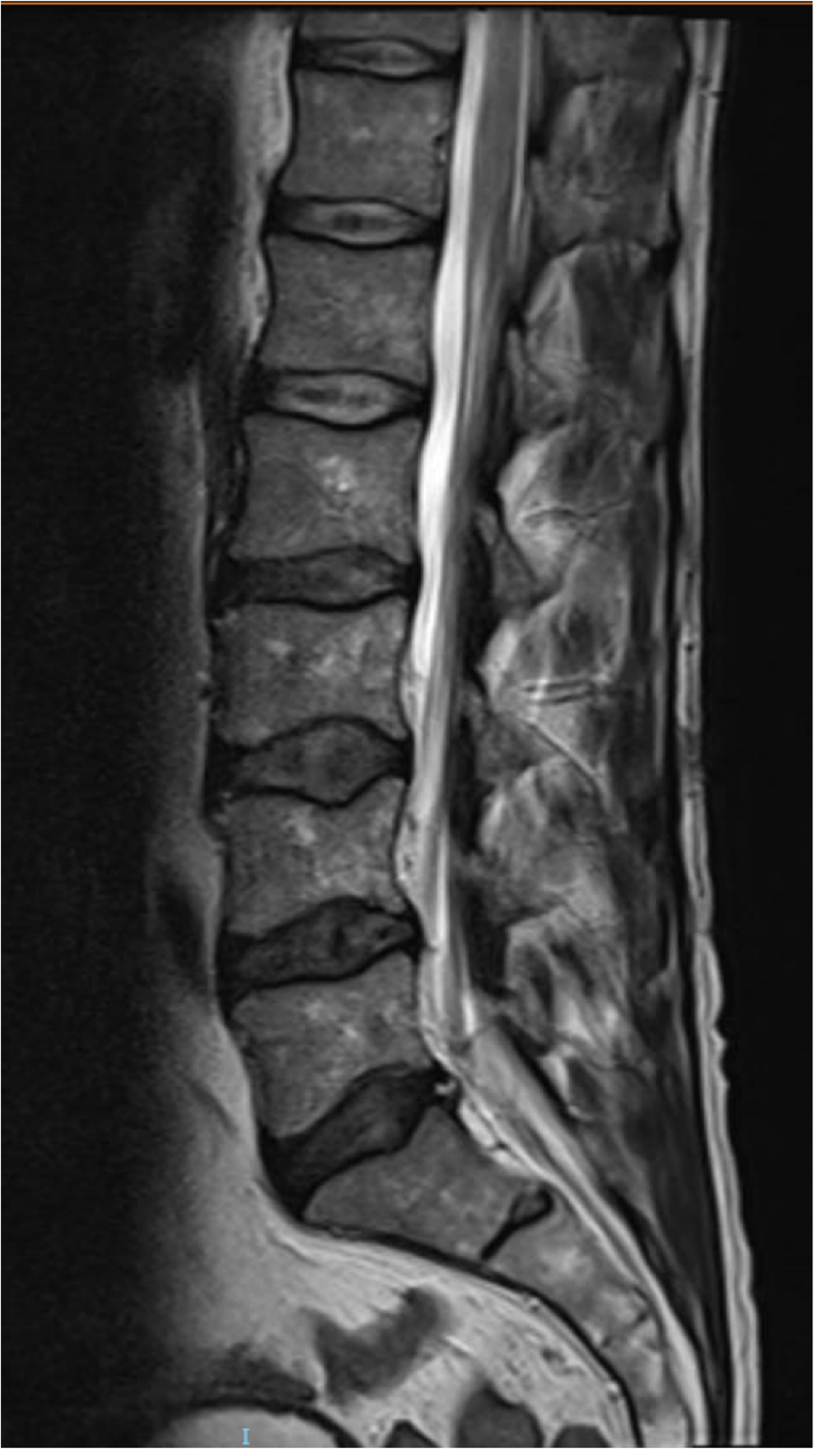
Magnetic resonance imaging of the lumbo-sacral spine showing no significant spinal cord compression

The presentation of lower back pain with bladder and bowel symptoms led to the emergency department (ED) team making an initial diagnosis of Bell’s palsy and cauda equina syndrome and arranging magnetic resonance imaging (MRI) of the lumbo-sacral spine. This demonstrated only a small disc protrusion at L5/S1 abutting the transiting right S1 root (Fig. 1).

**Fig. 2:**
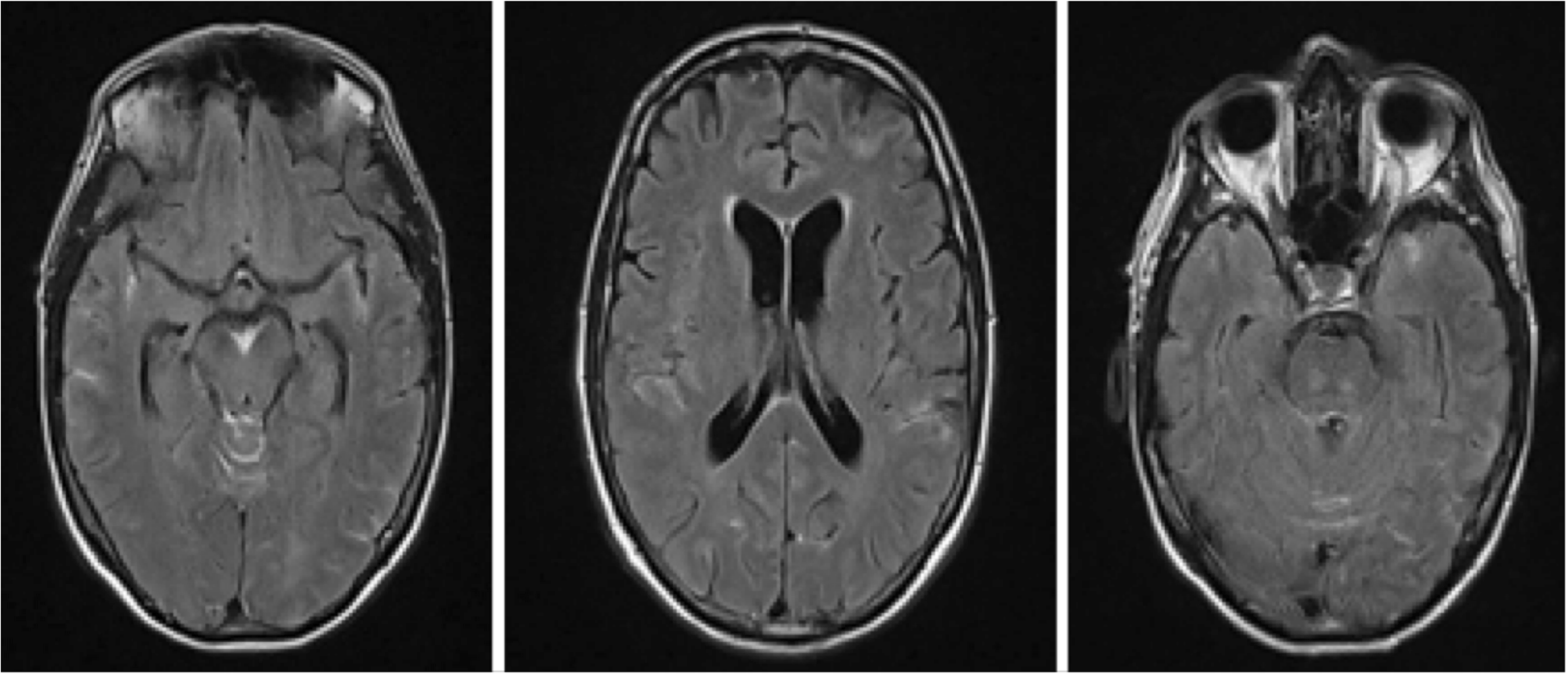
Magnetic resonance images of the brain showing diffuse subarachnoid fluid-attenuated inversion recovery (FLAIR) hyperintensity concerning for blood within the parafalcine, left frontal, bilateral occipital and bilateral temporal sulcal spaces, Sylvian fissures and the basal cistern. It also showed a focus of cortical/subcortical high signal in the left superior parietal lobule. No meningeal or parenchymal enhancement was demonstrated

**Fig. 3:**
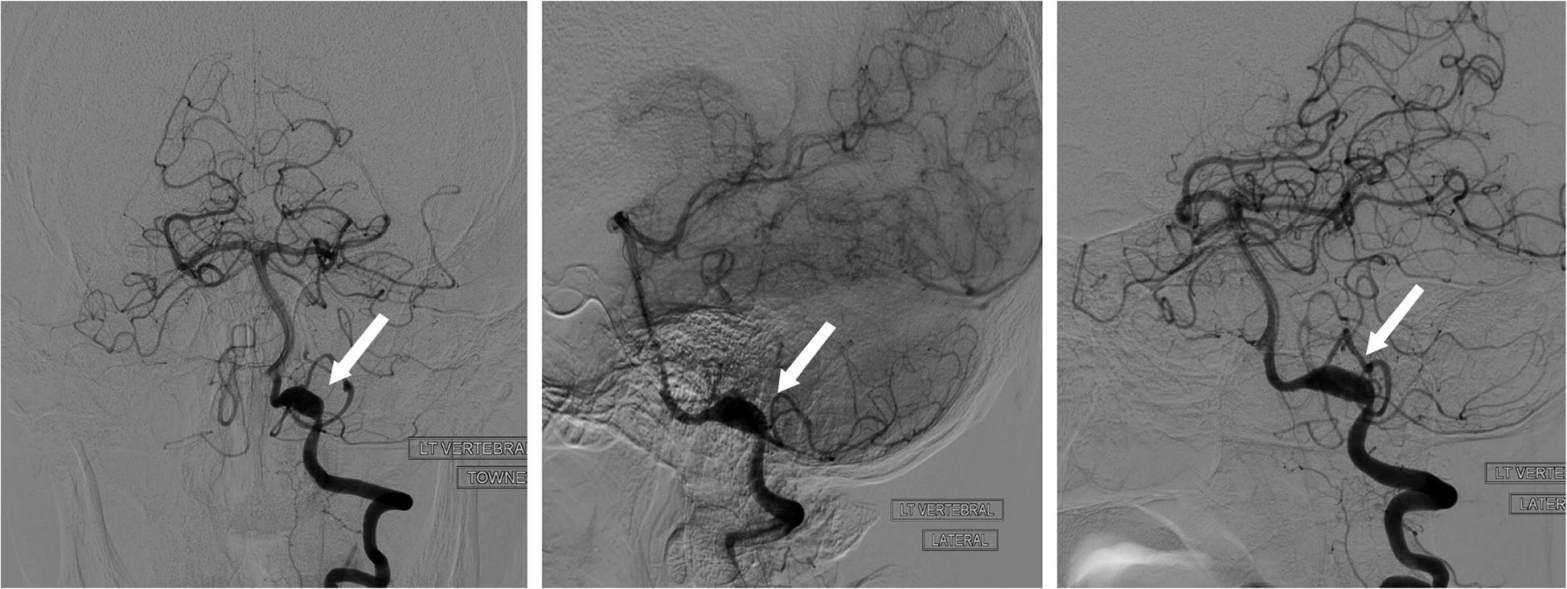
Digital subtraction angiography revealing fusiform dilatation of the left intradural vertebral artery (white arrow)

Due to the facial nerve palsy, imaging for a central cause was performed. MRI of the brain showed diffuse subarachnoid fluid-attenuated inversion recovery (FLAIR) hyperintensity concerning for blood, including in the basal cistern and Sylvian fissures. There was also a focus of cortical/subcortical high signal in the left superior parietal lobule (Fig. 2). MRI of the cervico-thoracic spine did not demonstrate SAH. The SAH was confirmed by computed tomography (CT). To investigate for a cause, CT angiography (CTA) was performed which demonstrated some prominence of the left-sided parafalcine cortical vessels. Digital subtraction angiography (DSA) demonstrated a left VAD (Fig. 3), with a dissection flap within a fusiform dilatation of the left intradural vertebral artery.

When SAH was confirmed, nimodipine 60 mg 4-hourly for 21 days, analgesia, euvolaemia and bed rest were commenced. Given the facial palsy, eye protection was ensured. The patient was discussed at the neurovascular multi-disciplinary team (MDT) meeting where conservative management of the VAD was advocated based on a delayed and atypical presentation, treatment risking vessel occlusion, and patient choice.

**Fig. 4:**
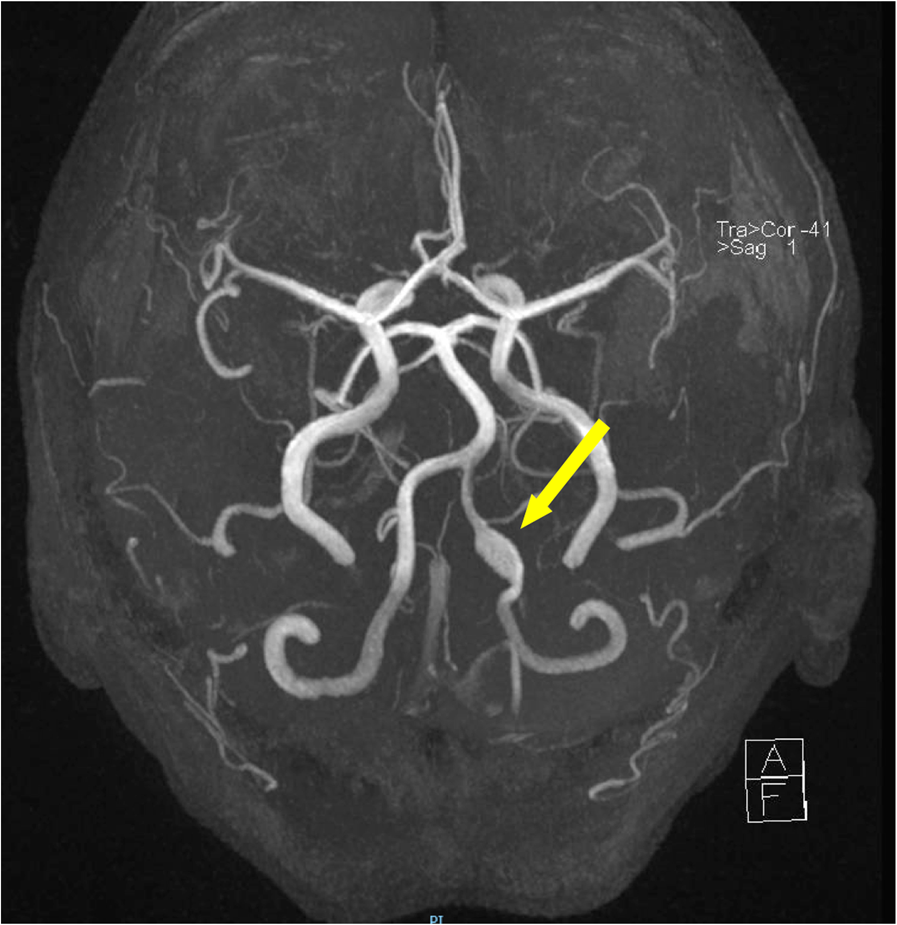
Magnetic resonance angiography 9-months post-presentation demonstrating stable appearances of the dissection aneurysm (yellow arrow)

The patient was monitored on the Intensive Care Unit following the DSA, and was later transferred to the neurosurgical ward. During the admission the patient had headache, intermittent confusion, and poor memory. Repeat CTA at 2 weeks showed stable appearances of the dissection and the patient was discharged with outpatient follow-up,

Clinic review at 12 weeks revealed the patient had returned to full-time work with resolution of most symptoms, apart from intermittent paraesthesiaes in the toes. Cranial nerves were intact with full recovery of facial nerve function, and peripheral neurological examination was normal. Magnetic resonance angiography (MRA) at 12 weeks showed stable appearances of the dissecting aneurysm, with no new parenchymal abnormalities nor other adverse features. Repeat MRA at 9 months demonstrated no change in the aneurysm. (Fig. 4) Imaging surveillance is to continue, with repeat MRA planned after a further 6 months.

## Discussion

We report a rare cause of VAD with SAH that initially presented with predominantly spinal symptoms. The presentation of SAH with cauda-equina features has been reported very rarely with SAH. In one case, arachnoiditis was the reported cause [7]. However, this is a delayed complication, which imaging excluded this in our case [8]. An alternative explanation is spinal cord ischaemia, which is very rarely associated with SAH [9, 10]. With cord ischaemia, image findings are delayed, [11] which has also been reported when cord infarction is due VAD, [12] and thus repeat spinal imaging would have been valuable in our case. An alternative consideration for cauda-equina like features is spinal SAH, which can cause back pain, sensory disturbance, and sphincter disturbance [13]. Very rarely, intracranial VAD has been associated with spinal extension of SAH [5, 6]. Although MRI in our case did not suggest a spinal SAH, MRI is not perfectly sensitive for spinal SAH, and in one case report MRI imaging of the spine was initially normal [14]. Moreover, the intensity of back pain in patients with SAH often correlates with the amount of blood in the lumbo-sacral subarachnoid space, [9] and the back pain had largely resolved by the time of admission in this case.

The initial absence of headache or neck stiffness in our case is unclear. It may reflect a combination of severe pain elsewhere (distraction) and confusion. It could also reflect that the headache developed due to redistribution of blood products. In a series of 8 patients with spinal SAH, 25% also had cortical/convexal SAH (cSAH) [13]. In our case, a convexal/subconvexal focus of blood affecting the parietal lobule was seen, which could explain why the patient developed headache, facial nerve palsy, and confusion. In various case series of cSAH, reported features have included focal and transient motor and/or sensory symptoms (42–73%), including facial nerve palsy and pronator drift [15, 16]; headache (18–65%); and confusion (9.8%) [17–20]. Although cSAH by definition spares the basal cistern and Sylvian fissures (which our case does not), descriptions of cSAH have been made even when these regions are not spared [21–23]. Alternative considerations for facial nerve palsy are compression by the aneurysm (which imaging did not suggest in our case) and vascular spasm disturbing the blood supply of the facial nucleus [24, 25].

There is no consensus for the treatment of intracranial dissections, [26] but treatment is usually performed because of the high risk of rebleeding within the first days of the event [27]. Endovascular techniques have been the mainstay of treatment, as these aneurysms are typically inaccessible surgically [28]. Conservative management with follow-up and blood pressure control is also an option, which is a decision determined by the assessment of risk for a poor outcome (such as poor neurological status on admission, rebleeding, and lesions with a pearl-and-string-structure) and the risk and feasibility of intervention [29]. Factors influencing our decision were the delayed and atypical presentation, the good neurological status on admission, patient aversion to operative management, and the feasibility of intervention - with the location of the fusiform aneurysm necessitating occlusion of the posterior inferior cerebellar artery. Flow diversion was also an off-label option, with limited data suggesting comparable outcomes in the long-term to conventional techniques, but with potentially greater short-term complications and the need for dual antiplatelet therapy [30].

## Conclusions

This case details a rare presentation of intracranial VAD with spinal symptoms, prior to the development of features that were more suggestive of a parietal focus of cortical blood. This case highlights the wide range of presenting features of SAH and the need for the consideration of SAH for sudden-onset spinal symptoms.

## Data Availability

Not applicable.

## Abbreviations

cSAH: cortical/convexal subarachnoid haemorrhage
CT: Computed tomography
CTA: Computed tomography angiography
DSA: Digital subtraction angiography
FLAIR: Fluid-attenuated inversion recovery
MDT: Multidisciplinary team
MRA: Magnetic resonance angiography
MRI: Magnetic resonance imaging
SAH: Subarachnoid haemorrhage
VAD: Vertebral artery dissection
